# A real-time arbitrary-shape text detector

**DOI:** 10.1371/journal.pone.0302234

**Published:** 2024-04-16

**Authors:** Manhuai Lu, Langlang Li, Chin-Ling Chen

**Affiliations:** 1 College of Mechanical and Electrical Engineering, University of Electronic Science and Technology of China, Zhongshan Institute, Zhongshan, China; 2 School of Mechanical and Electrical Engineering, University of Electronic Science and Technology of China, Chengdu, China; 3 School of Information Engineering, Changchun Sci-Tech University, Changchun, China; 4 Department of Computer Science and Information Engineering, Chaoyang University of Technology, Taichung, Taiwan; Nanchang University, CHINA

## Abstract

It is challenging to detect arbitrary-shape text accurately and effectively in natural scenes. While many methods have been implemented for arbitrary-shape text detection, most cannot achieve real-time detection or meet practical needs. In this work, we propose a YOLOv6-based detector that can effectively implement arbitrary-shape text detection and achieve real-time detection. We include two additional branches in the neck part of the YOLOv6 network to adapt the network to text detection, and the output side uses the pixel aggregation (PA) algorithm to decouple the PA output to use it as the detection head of the model. Experiments on benchmark Total-Text, CTW1500, ICDAR2015, and MSRA-TD500 showed that the proposed method outperformed competing methods in terms of detection accuracy and running time. Specifically, our method achieved an F-measure of 84.1% at 291.8 FPS for 640 × 640 Total-Text images and an F-measure of 81.5% at 199.6 FPS for 896 × 896 ICDAR2015 incidental text images.

## Introduction

The recent progress in intelligence and digitization has made natural scene text reading more attractive [1–3]. Scene text detection and recognition technology are widely used in intelligent systems, such as automatic driving systems, visual impairment assistance systems, and smart robots. Moreover, scene text, such as road signs, store signs, and product packaging, is ubiquitous daily. However, the various shapes and sizes of scene text objects pose challenges for detection and recognition [4, 5]. Text detection, a prerequisite for text recognition in the whole detection and recognition system. Researchers have explored horizontal, directional, and arbitrary-shape text [4]. However, detecting arbitrary-shape text still faces significant challenges owing to the diversity of text shapes, which causes difficulties in the design of detection algorithms. For example, the detection box must adapt to texts with different forms, ensure tightness between the text detection box and the text region, avoid introducing background noise, and maintain a certain detection speed. Some methods can accurately represent text regions, but the algorithm’s complexity slows down detection [6, 7]. Some methods achieve real-time speed in detection but do not break through the limitations of text shape [8]. Therefore, the challenge of arbitrary-shape text detection is gaining a better trade-off between detection accuracy and speed while ensuring tightness between the detection box and the text region. The two main reasons for this difficulty are that for text objects of different sizes and shapes, the detection network must have a robust feature extraction capability to detect text objects of various sizes simultaneously, which increases the computational cost of the model to a certain extent. Besides, the generation and representation of detection boxes need to satisfy flexibility, and the text bounding boxes must fit To solve the problems of low detection speed and difficulty of arbitrary-shape text detection, we propose a real-time arbitrary-shape text detector.

Inspired by YOLOv6 [9], we improved it to enhance text detection speed for scene text detection. We propose using an improved network based on YOLOv6 to extract features and a detection part based on a decoupled output head using pixel aggregation (PA) [10] to achieve detection. Owing to the flexibility of the PA algorithm, we use it in the detection head to detect arbitrary-shape text, so the final generated detection box can fit the text region well. The improved detection network has good adaptability with the PA-based decoupled head, enabling better performance in arbitrary-shape text detection while ensuring a certain detection speed.

All of our contributions are as follows: 1) We have added two branches to the YOLOv6 network to make it more applicable to the characteristics of text objects. 2) We perform a decoupling operation on the output of the detection head, separating the text kernel and text region as one part and decoupling the instance vector as another part to improve the detection performance of the model. The detection head can directly output text bounding boxes. 3) We use a loss function weighted by dice loss and focal loss to control the generation of text regions, which effectively improves the model training performance. Eventually, the model can achieve arbitrary-shape text detection, be trained end-to-end, accomplish a similar detection accuracy as the previous model, and surpass their inference speed.

The rest of the paper is structured as follows. The Related works section briefly reviews related work on scene text detection. The Methods section describes our proposed method in detail. We present the experimental steps and results in Experiments section. The Discussion section discusses the experimental results. Finally, Conclusion section concludes this paper with potential future research directions.

## Related works

Object detection is a fundamental problem in computer vision, and text objects have certain specific characteristics compared with general detection objects. Therefore, object detection models provide some inspiration for text detection algorithms. Many networks for text detection are based on object detection networks, and feature pyramid network (FPN) [11] and residual connections are widely used in text detection to enhance network depth and feature extraction capabilities. Similarly to YOLOv4 [12] and YOLOv5 [13], YOLOv6 uses a network structure designed based on the pixel aggregation network (PAN) topology [14], which adds bottom-up path augmentation to the FPN cascade structure to shorten the information path between lower and highest layers and facilitate information flow. YOLOv6 has a backbone network based on the method of structural re-parameterization in RepVGG [15], fully utilizing hardware computing power to enhance network representation while reducing inference latency. Therefore, the YOLOv6 network structure has certain advantages in inference speed, providing a direction to improve detection speed.

Some important works in deep learning has significant research implications. [16] expands samples by introducing interference of different intensities into the spectrum of radio signals, which use bidirectional noise masks that meet specific distributions to achieve interference on the spectrum. This method brings effective data augmentation methods and is of great significance. Zheng et al. [17] applied manifold regularization to autoencoders to promote cross-layer manifold invariance. Ms-RaT [18] designed a dual-channel representation (DcR) of radio signals to help the model learn discriminative features by converging the multi-modality information. Zheng et al. [19] developed a multidimensional LSTM that simultaneously considers spatial and temporal information. Multi -scale analysis of temporal sequence is of great help in improving cross-regional generalization of deep learning models, which is significant for deep learning research. [20] proposed a prior regularization method in deep learning to guide loss optimization during model training. This method preserves the original signal information as much as possible while fully utilizing prior knowledge, which helps to improve the generalization of deep learning models on signals with various signal-to-noise ratios.

Two mainstream text detection methods exist: regression-based and segmentation-based methods.

Regression-based methods mainly use a top-down approach for text detection, whether through direct or indirect regression, by regressing text boxes to achieve detection. For arbitrary-shape text, the representation of the text box increases the difficulty of regression owing to its shape variability. Some researchers have improved the effectiveness of text detection by optimizing the representation of the text detection box. DMPNet [21] uses tight quadrilateral bounding boxes to detect text, reducing background noise introduced by poor tightness between horizontal rectangular bounding boxes and text regions. Moreover, the backbone network of EAST [22] uses PVANet [23] and adds a cascaded feature fusion branch for text detection. Text boxes are represented by rotated rectangular boxes and quadrilateral bounding boxes, which can detect directional and quadrilateral text. Zhang et al. [24] proposed a feature enhancement network (FEN) for region proposal and text detection optimization. FEN combines high-level semantic features with low-level semantic features to enhance model detection performance. The final detection box is represented by a rectangle. This fusion of high-level and low-level features can effectively improve network detection performance.

TextBoxes, proposed by Liao et al. [25], is a 28-layer fully convolutional network with a text-box layer designed at the output-to-output features of different scales. The detection box adds an offset in the vertical direction to increase the fit of the rectangular box to the text region. To overcome the limitation of only detecting horizontal text, TextBoxes++ [26] introduces rotated rectangles and quadrilateral bounding boxes based on TextBoxes to adapt to seeing directional text. For irregular text detection, Liu et al. [6] proposed the adaptive Bezier-curve network (ABCNet) for rare text detection. ABCNet fits directional or curved text with parametric Bezier curves and introduces only a small computational load. Using Bezier curves to express text boxes has also been widely used in other studies [27–29]. Bezier curves significantly enhance text detection’s flexibility, but their computational process is not conducive to high detection speed.

Regression-based methods have difficulty with arbitrary-shape text detection to text detection box limitations. Researchers have improved text box representation to adapt to text shape changes, but detection remains difficult for text with complex shapes and introduces additional computational costs, which reduce detection speed.

Segmentation-based approaches are bottom-up to detect text regions, and they use semantic segmentation to convert the text detection problem into a classification problem. Some methods segment text regions into connected components and then connect them to represent text regions [30–32]. This approach is highly flexible and facilitates arbitrary-shape text detection. For example, the pixel link proposed by Deng et al. [30] segments text instances by connecting pixels in the same text instance, and text bounding boxes are directly extracted from the segmentation results. Similarly, TextSnake [31] describes the text as an ordered and overlapping sequence of discs centered on a symmetric axis, with each disc having the two properties of radius and direction to adapt to changes in text shape, allowing the method to represent the text of any shape flexibly. TextDragon [32] describes text shape through a series of quadrilaterals, first segmenting the text region into a series of local quadrilaterals and then connecting them to obtain detection results. This local-to-whole idea overcomes the limitation of text box shapes in regression-based methods, but some post-processing operations need to be added to generate text boxes.

Furthermore, some researchers have reduced the complexity of post-processing by optimizing segmentation algorithms. DBNet [33], for example, can perform binarization operations in segmentation networks, and the differentiable binarization (DB) module enables the segmentation network to set thresholds for binarization adaptively. The DB module not only simplifies post-processing operations but also improves the performance of the text detector. Dai et al. [34] implemented multidirectional scene text detection through instance-aware semantic segmentation. The proposed fused text segmentation network exploits the advantages of both semantic segmentation and region-based target detection tasks by combining multilevel features. Tian et al. [35] mapped text pixels onto an embedding space, where pixels belonging to the same text are stimulated to approach each other gradually. In this way, the segmentation of text regions is directly accomplished. These methods achieve text region segmentation from unique perspectives but may lack generalizability.

Wang et al. [36] proposed the progressive scale expansion network (PSE-Net) for pixel-wise segmentation, which can detect text of arbitrary shapes. To improve the detection efficiency of PSE-Net and PAN, Wang et al. [10] proposed the feature pyramid enhancement module and the feature fusion module, effectively reducing computational consumption. A learnable post-processing operation, PA, was also presented to accurately aggregate text pixels based on predicted similarity vectors to obtain the final text box. The PA algorithm can well represent text regions, has good generalizability, and has no additional computational consumption, making it suitable for arbitrary-shape text detection. Segmentation-based approaches benefit from the flexibility of their text region representation, which is not limited by text shape but can be equally limited by algorithm complexity. Therefore, researchers have continuously optimized segmentation algorithms to reduce the complexity of post-processing and improve the robustness and efficiency of detection models. One of the development trends of scene text detection is to improve model robustness and detection speed while ensuring a certain level of detection accuracy. Considering detection speed and flexibility, we combine the improved YOLOv6 network structure with the PA post-processing algorithm to achieve real-time detection of arbitrary-shape text.

## Methods

**Fig 1:**
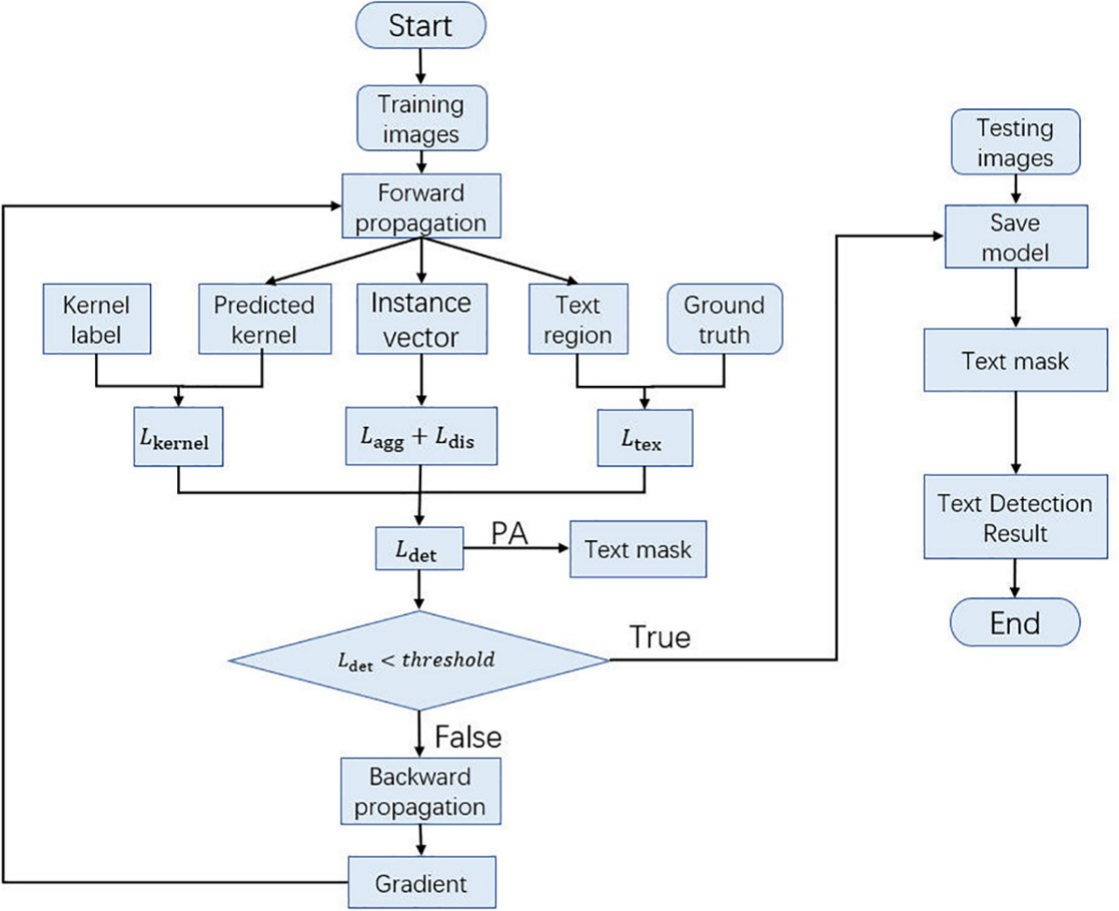
The flow chart of scene text detection.

In this section, the details of our proposed method are described. The flow chart of the model detection is shown in Fig 1. The training images are first propagated forward to predict the text region and kernel masks. Then, the loss function continuously corrects the prediction results to generate the text mask by the PA algorithm. Finally, the final text detection results are generated based on the predicted text mask.

### Network architecture

The whole network is divided into the backbone, neck, and head. As shown in Fig 3, the backbone and neck of the network are both re-parameterizable structures. We draw five branches from the backbone, and the branches use a composite structure of FPN and PAN in the neck part, forming a five-scale network feature representation. The FPN enhances the feature extraction ability of the network, and the PAN structure promotes the information flow, effectively improving network efficiency. The detection head of the network is shown in Fig 4. The feature map obtained from the neck can output five sets of instance vectors, text regions, and text kernels of different scales in the head. We first decouple them to output, then fuse the features of five groups of feature maps with different scales into one set of output, and finally use the PA algorithm to obtain a text mask. The text bounding box is obtained from the segmentation result. This section introduces the details of each part of the network structure in detail.

#### Backbone network

RepVGG [15] uses a structure re-parameterization technique to decouple multibranch structures (in training time) and single-branch structures (in inference time), and structure re-parameterization is implemented by converting the parameters of one structure into the parameters of another structure. Because the multibranch structure is beneficial to alleviate the problem of gradient disappearance, and there is no gradient transfer in the inference phase, the single-branch structure is more advantageous during inference. Decoupling the network structure of the training and inference processes by structural re-parameterization reduces the computational consumption of RepVGG and enables it to outperform the VGG network on ImageNet.

**Fig 2:**
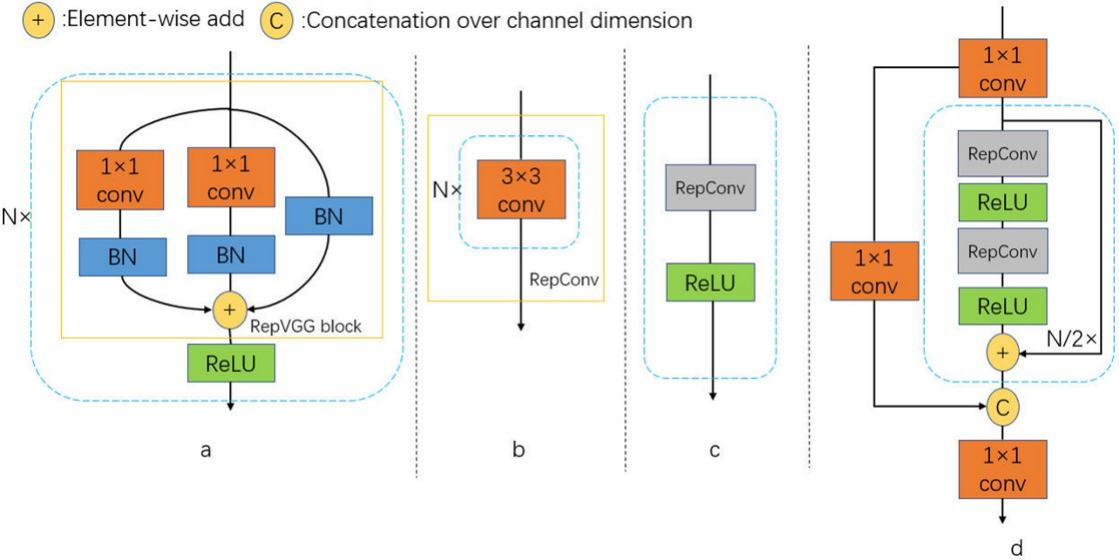
Structure of the blocks. (a) At training time, RepBlock comprises a stack of RepVGG blocks with ReLU activations. (b) RepConv consists of a stack of 3 × 3 convolutional layers. (c) At inference time, the RepVGG block is converted to RepConv. (d) CSPStackRep Block consists of three 1 × 1 convolutional layers and two subblocks composed of RepConv and ReLU with the residual connection.

YOLOv6 uses the idea of re-parameterization to design the parameterizable backbone structure EfficientRep [9]. In the small model, the main structure of the backbone network in the training phase is the Repblock [9], which is a stack of RepVGGblocks [9], as shown in Fig 2(a). During the inference stage, the Repblock is transformed into a stack of 3 × 3 convolutional layers (named Repconv) with a combination of RELU activation functions, as shown in Fig 2(c).

Large models of YOLOv6 have deeper network layers, stronger feature extraction ability, and the best performance in text detection. Large models also build the backbone using the CSPStackRep Block [9]. CSPStackRep Block is composed of three 1 × 1 convolution layers and a stack of subblocks consisting of two RepVGG blocks (converted to RepConv in the inference phase) with a residual connection, as shown in Fig 2(d). Structural re-parameterization converts the RepVGG blocks (in training time) in CSPStackRep Block to RepConv (in inference time) to achieve structural transformation for a better computational load and accuracy trade-off.

#### Rep-PAN with five branches

The neck of YOLOv6 uses FPN and a PAN topology structure [14], Rep-PAN, which is similar to YOLOv4 [12] and YOLOv5 [12] with a CSP [37] structure, replacing the CSPBlock in YOLOv5 with a CSPStackRep block and changing the width and depth [9].

**Fig 3:**
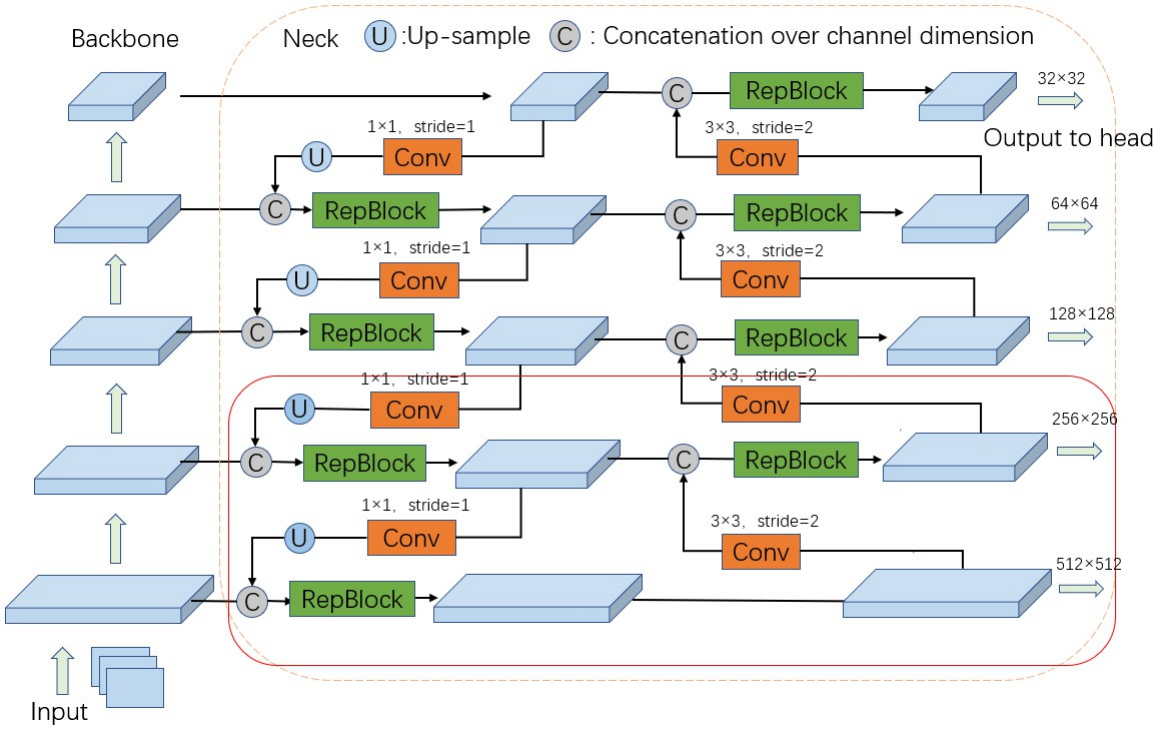
Structure of the backbone and neck.

Unlike the neck in YOLOv6, where three branches are elicited from the backbone, we include two branches to make the detection network adapt to the characteristics of text objects and improve the performance of the model in text detection, as shown in Fig 3. The original three branches are elicited in the last three blocks of the backbone, which are insufficient for small object extraction. In object detection, high-level features contain rich semantic information, which can be used to detect large targets, but the object location is coarse. However, low-level features have less semantic information, and the object location is precise, which is suitable for detecting smaller targets. Compared with general objects, text objects are more complex and unique. We introduce branches after each of the five blocks of the backbone to improve the detection ability of the model for small text. The five branches are input to the neck, and the final feature map is output to the detection head after the structure of FPN and PAN. The structure of FPN and PAN promotes the information interaction between feature maps and effectively improves the feature extraction ability of the network.

While adding a certain amount of computation and computational memory, our improvement significantly improves the detection capability of the network, especially for smaller text objects, making the network more adaptable to the characteristics of text objects, such as different sizes and unique shapes.

#### Decoupled head-based PA

ur model uses the PA [10] algorithm to output three parts: text regions, kernel and instance vectors. The text region represents the complete shape of a text line, but the text regions that are close to each other may overlap. The text kernel is formed by inwardly shrinking a portion of the text region. Thus, the text kernel can distinguish adjacent text regions well, but cannot represent the complete text shape. Instance vectors are a high-dimensional representation of information, containing instance information about each pixel. Instance vectors can express the similarity of pixels, where pixels belonging to the same text line have similar instance vectors. Therefore, instance vectors can categorize pixels in a text region into corresponding text kernels and successfully associate text kernels with text regions.

**Fig 4:**
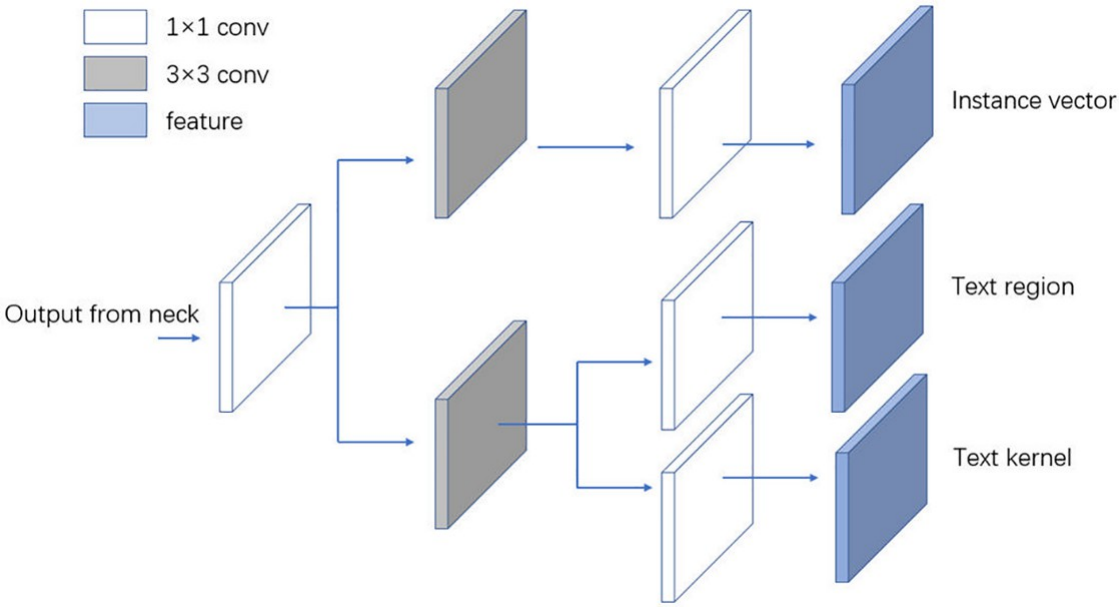
The decoupled output of instance vector, text region, and text kernel.

Inspired by the literature [38–40], we decouple the three parts of the output as shown in Fig 4. Wu et al. [39] argued that the classification and regression tasks belong to different types of tasks in object detection. Therefore, improving model performance by decoupling the classification task from the regression task has been widely used in many one- or two-stage models [39, 41]. We consider that the three outputs of the PA algorithm—text regions and text kernels, which can both address the shape representation of text regions, and instance vectors, which address the similarity of pixels in text regions—also belong to different types of tasks. Therefore, the three outputs can be decoupled into two output branches. We output the text region and text kernel in one branch and the instance vector in the other.

The details of decoupling are as follows. As shown in Fig 4, we first use a 1 × 1 convolution to reduce the dimension of the features of the Neck output. Then, we use a 3 × 3 convolution to extract two parallel branches, outputting the instance vector as one branch. The second branch outputs the text region and text kernel. We use only one 3 × 3 convolution in each decoupling branch to reduce computational consumption. After decoupling the output of the PA algorithm, we find that this effectively improves the detection performance of the model.

The output of the Neck has five output branches with different scales. After decoupling, the head part also outputs five sets of feature maps with different scales (each set contains three feature maps: instance vector, text region, and text kernel). We fuse the feature maps with different scales to obtain the final output. The three outputs compensate for each other so that the detection model can accurately detect arbitrary-shape text. The instance vector, text region, and text kernel are used to generate the final text mask by the PA algorithm, and the last text bounding box can be obtained from the text mask. Note that our feature fusion is performed separately for each output, and the instance vector, text region, and text kernel are fused, respectively. The fusion is performed by up-sampling five groups of features with different scales into the same scale and then averaging the feature maps to obtain the fused feature maps.

### Loss function

The loss function of text detection can be expressed as

$$\begin{array}{c} L_{\text{det}}=L_{tex}+\alpha L_{\text{ker}}+\beta L_{pa} \end{array}$$ (1)

$$\begin{array}{c} L_{pa}=L_{agg}+L_{dis} \end{array}$$ (2)

where *L*_*tex*_ is the text region segmentation loss function, *L*_*ker*_ is the text kernel segmentation loss function, and *L*_*pa*_ is the PA algorithm loss function. Here, pixel aggregating and pixel separating are controlled by *L*_*agg*_, *L*_*dis*_ respectively. *α* and *β* are used to balance the weight of *L*_*tex*_, *L*_*ker*_, and *L*_*pa*_ in the detection loss. Here, *α* is set to 0.5, and *β* is set to 0.25.

Considering that the text area is much smaller than the non-text area in the text object and there is a sample imbalance problem, we use dice loss [42] and focal loss [43] as the loss functions to optimize the segmentation results. Dice loss and focal loss are both loss functions for the category imbalance problem. For the text region segmentation loss function *L*_*tex*_, we weigh the combination of dice loss and focal loss and use the focal loss to make the training process smoother, allowing the model to predict text regions more gently and promote training convergence. Since the text kernel labels are obtained by shrinking the text region, the text kernel is smaller than the text region, which increases the number of small objects to some extent. The imbalance problem when predicting the text kernel is more serious than in the text region. For the text kernel segmentation loss function, we only use dice loss as the loss function to reduce the effect of extreme sample imbalance. *L*_*tex*_ and *L*_*ker*_ are formulated as follows:

$$\begin{array}{c} \begin{array}{cc} & L_{text}=\left(1-\lambda \right)\times [1-\frac{2\sum_{i}P_{tex}\left(i\right)G_{tex}\left(i\right)}{\sum_{i}P_{tex}{\left(i\right)}^{2}+\sum_{i}G_{tex}{\left(i\right)}^{2}}]+\\ & \lambda \times [(-\alpha {\left(1-p\right)}^{\gamma }y\cdot \text{log}\left(p\right))-\left(1-\alpha \right)p^{\gamma }\left(1-y\right)\cdot \text{log}\left(1-p\right)] \end{array} \end{array}$$ (3)

$$\begin{array}{c} L_{ker}=1-\frac{2\sum_{i}P_{\text{ker}}\left(i\right)G_{\text{ker}}\left(i\right)}{\sum_{i}P_{\text{ker}}{\left(i\right)}^{2}+G_{\text{ker}}{\left(i\right)}^{2}} \end{array}$$ (4)

Here, *P*_*tex*_(*i*), *G*_*tex*_(*i*) denote the segmentation result and the i-th pixel value of the true frame of the text region, respectively, and *P*_*ker*_(*i*), *G*_*ker*_(*i*) denote the prediction of the text kernel and the i-th pixel value of the true bounding box, respectively. *λ* is set to 0.5 in *L*_*tex*_, *α* is set to 0.25, and *γ* is set to 2 in the Focal loss function. Here, p denotes the probability that the current pixel is a positive sample, taking values from 0 to 1; y denotes the true value, either 0 or 1. We also employ OHEM [44] to ignore those simple non-text pixels in the calculation of *L*_*tex*_. As in [10], the loss functions *L*_*agg*_ and *L*_*dis*_ of the PA algorithm are calculated as follows:

$$\begin{array}{c} L_{agg}=\frac{1}{N}\sum_{i=1}^{N}\frac{1}{\left|T_{i}\right|}\sum_{p\in T_{i}}D_{1}\left(p,K_{i}\right) \end{array}$$ (5)

$$\begin{array}{c} D_{1}\left(p,K_{i}\right)=\text{ln}\left(R{\left(||F\left(p\right)-g\left(K_{i}\right)||-\delta_{agg}\right)}^{2}+1\right) \end{array}$$ (6)

where N denotes the number of text lines, *T*_*i*_ denotes the text region of the ith text line; *K*_*i*_ denotes the text kernel of the text line *T*_*i*_; *D*_1_(*p*, *K*_*i*_) denotes the distance between the text pixel p and the text kernel *K*_*i*_; R(·) denotes the ReLU function to ensure that the output is non-negative; F(p) denotes the instance vector of pixel p; *g*(*K*_*i*_) denotes the instance vector of the text kernel *K*_*i*_, which can be calculated by the formula $g\left(K_{i}\right)=\sum_{p\in K_{i}}F(p)/\left|K_{i}\right|$; *δ*_*agg*_ is a constant, which is set to 0.5 here. The function of *L*_*agg*_ is to cluster the pixels of a text line into its corresponding text kernel and achieve aggregation by minimizing the distance between the pixels belonging to the same text line and the text kernels. To distinguish different text lines, we must establish a certain distance between the text kernels of different text lines. Therefore, the instance vector of a text kernel also must have a certain distance from another text kernel’s instance vector. Moreover, the instance vector of a text kernel must have a certain distance from the instance vector of a background pixel to distinguish the background pixel. This can be achieved by *L*_*dis*_.

$$\begin{array}{c} L_{dis}=\frac{1}{N^{2}}\overset{N}{\underset{i=1}{\sum }}\left(D_{b}\left(K_{i}\right)+\overset{N}{\underset{\begin{array}{l} i=1\\ j\neq i \end{array}}{\sum }}D_{2}\left(K_{i},K_{j}\right)\right) \end{array}$$ (7)

$$\begin{array}{c} D_{b}\left(K_{i}\right)=\frac{1}{\left|B\right|}\sum_{p\in B}\text{ln}(R{\left(\delta_{dis}-||F\left(p\right)-g\left(K_{i}\right)||\right)}^{2}+1) \end{array}$$ (8)

$$\begin{array}{c} D_{2}\left(K_{i},K_{j}\right)=\text{ln}\left(R{\left(\delta_{dis}-||g\left(K_{i}\right)-g\left(K_{j}\right)||\right)}^{2}+1\right) \end{array}$$ (9)

Here, B denotes the background, *D*_*b*_(*K*_*i*_) denotes the distance between the text kernel *K*_*i*_ and background pixels; *D*_2_(*K*_*i*_, *K*_*j*_) represents the distance between the text kernel *K*_*i*_ and text kernel *K*_*j*_; *δ*_*dis*_ is a constant, which is set to 3 here. The role of *L*_*dis*_ is to separate text kernels of different text lines and to separate text kernels from background pixels. In the testing phase, the predicted instance vector guides pixels belonging to the same text line to converge to their corresponding text kernel. The PA process of the PA algorithm consists of three parts: (1) finding the text core region from the segmentation results; (2) for each text kernel *K*_*i*_, aggregating the pixels in its pixel-4 field until the Euclidean distance between the corresponding pixel and the instance vector of the text kernel is less than a threshold d; (3) repeating the second step until no pixel satisfying the condition is acquired in the text region. The flexibility and generalization of the PA algorithm enable our detection head to adapt well to the detection network and to achieve end-to-end training and satisfactory results.

## Experiments

We conducted a series of experiments to demonstrate the validity of our model. Our experiments were conducted on a Windows operating system using a single GPU RTX3090, configured with CUDA and CUDNN environments to enable GPU-accelerated computation on the data. The deep learning framework was built using PyTorch, using Python as the development language and Adam as the optimizer. We conducted experiments on the benchmark, widely used in text detection fields.

### Datasets

SynthText [45] is a large-scale synthetic dataset with 850,000 images, containing many multidirectional text lines with word-level and character-level bounding box annotations and text recognition content annotations. This dataset was used to pretrain our model.

Total-Text [46] is a dataset for arbitrary-shape text detection and recognition, containing horizontal text, tilted text, and curved text lines. Most of the text is in English, although a small amount is in Chinese. The dataset contains 1,225 training images and 300 test images. The text box annotation is in the form of word-level polygon annotations and transcriptions.

CTW1500 [47] is a dataset applied to arbitrary-shape text detection, containing 1,000 training images and 500 test images. The text annotation is in the form of polygon annotations at the text line level.

MSRA-TD500 [48] is a multidirectional text dataset, containing 300 training images and 200 test images, with text content in Chinese and English. The text boxes are labeled in the form of line-level rotated rectangular boxes. Owing to the small amount of data in the training set, we added 400 self-photographed multidirectional text images for model training, among which there are more Chinese texts.

ICDAR 2015 [49] is taken by Google Glass that does not focus on viewpoint and image quality. IC15 has 1500 images, of which 1000 are training images and the remaining 500 are test images. The text bounding box is determined by the four vertices of the quadrilateral and also labeled with word-level text transcriptions.

### Experiments on curved text datasets

#### Experiment settings

To test the effectiveness of the model in detecting arbitrary-shape texts, we conducted experiments on the Total-text and CTW1500 datasets, setting the instance vector dimension to 4, the distance threshold of PA to 3, the shrinking rate of the text kernel to 0.8, and the negative–positive ratio of OHEM to 3. Data enhancement methods used during training included random scaling, random horizontal flip, random rotation, and random clipping, and the image size was 640 × 640. All experiments were trained on a single GPU using the Adam [50] optimizer with a batch size of 4. Similar to other researchers [51], we used a polynomial learning rate adjustment strategy with the initial learning rate set to 1 × 10-3, and we multiplied the initial learning rate by ${\left(1-\frac{iter}{\text{max}\;iter}\right)}^{power}$ to achieve learning rate tuning, where power was set to 0.9 in all experiments. All results were tested in a single thread on an RTX3090 GPU with a batch size of 1. We used two training strategies to train our model: (1) We used external datasets to pretrain and then fine-tune on the dataset using the pretrained model, following [36, 52, 53]; (2) we trained directly on the existing dataset without using an external dataset, following the strategy in literature [22, 52, 54]. In the first strategy, we only trained one epoch on Synthtext and fine-tuned 150 epochs on curved text datasets. In the second strategy, we trained 300 epochs directly on the curved text dataset.

#### Curved text detection results

Seglink, TextSnake, and TextDragon can all detect irregular text, but their performance is poor when the distance between texts is far. LOMO can detect curved text, but it requires iterative refinement, which introduces a certain amount of computation. Our method can detect arbitrary-shape text well without additional calculation modules. As shown in Table 1, without external data pretraining, our model achieved an F-measure of 78.5% on the Total-Text dataset, outperforming many models, including some pretrained with SynthText, such as TextSnake and FOTS. Moreover, our method’s inference speed was seven times faster than PAN++ (the fastest among the comparison models). When trained using external datasets, the model achieved an F-measure of 84.1%, outperforming most models.

**Table 1:** Text detection results on Total-Text and CTW1500. * indicates the results from [28], + indicates the results from [42], ◊ indicates the results from [29]. FPS is tested on RTX3090.

Method	External Dataset	Total-Text				CTW1500			
		Precision	Recall	F-measure	FPS	Precision	Recall	F-measure	FPS
Seglink [53]	×	30.3*	23.8*	26.7*	-	42.3^+^	40.0^+^	40.8^+^	10.7^+^
EAST [22]	×	50.0*	36.2*	42.0*	-	78.7^+^	49.1^+^	60.4^+^	21.2^+^
DMPNet [21]	×	-	-	-	-	69.9^◊^	56.0^◊^	62.2^◊^	-
DeconvNet [55]	×	33	40	36	-	-	-	-	-
CTD+TLOC [47]	×	-	-	-	-	77.4	69.8	73.4	13.3
PAN++ [56]	×	89.2	80.3	84.5	38.3	85.2	81.1	83.1	36
**Ours**	×	**90.6**	69.1	78.5	**291.8**	88	61.5	72.4	**193.4**
TextSnake [31]	SynthText	82.7	74.5	78.4	12.4	67.9	85.3	75.6	-
TextDragon [32]	SynthText	85.6	75.7	80.3	-	84.5	82.8	83.6	-
FOTS [57]	SynthText	52.3	38.0	44.0	-	79.5	52.0	62.8	-
LOMO [58]	SynthText	88.6	75.7	81.6	4.4	89.2	69.6	78.4	-
CRAFT [59]	SynthText	87.6	79.9	83.6	4.8	86	81.1	83.5	7.6
PAN++ [56]	SynthText	89.9	81	85.3	38.3	87.1	81.1	84	36
**Ours**	SynthText	**92.5**	77.1	84.1	**291.8**	88.6	74.8	81.1	**193.4**

Without pretraining with external datasets, the model achieved an F-measure of 72.4% on the CTW1500 dataset, exceeding most previous methods. The model’s inference speed reached four times that of PAN++, surpassing all comparative models. After pretraining with SynthText, the F-measure of the model improved to 81.1%, surpassing FOTS, TextSnake, and LOMO.

**Fig 5:**
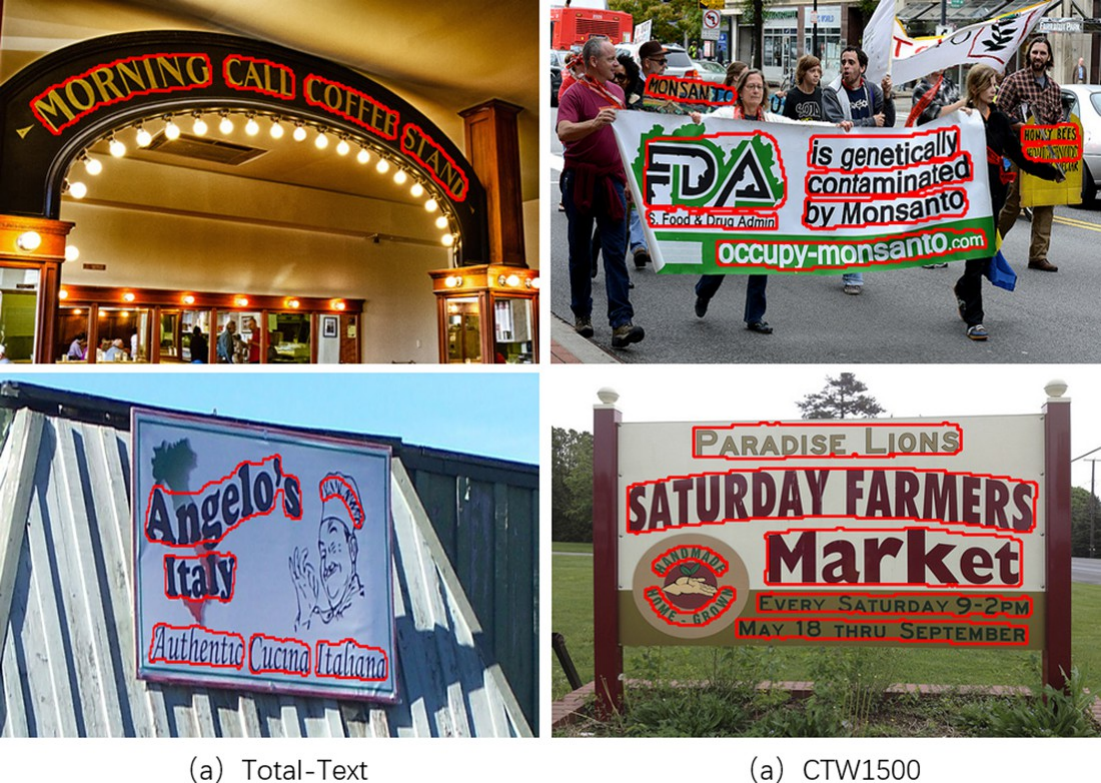
Detection results on Total-Text and CTW1500.

The results show that our model was competitive regarding the accuracy and speed of arbitrary-shape text detection. Some detection results are shown in Fig 5. Our model could effectively detect text with complex shapes.

### Experiments on straight text datasets

#### Experiment details

To verify the model’s effectiveness for directional text detection, we evaluated our model on IC15, a representative benchmark for text detection and text recognition tasks, and MSRA-TD500, commonly used for extended text detection. We set the shrinking rate r to 0.5 and 0.8 for IC15 and MSRA-TD500, respectively. Other parameter settings, data enhancement, and training schemes were consistent with the settings described in curved text detection. In our experiments, the image size was 896 × 896 in training and testing.

#### Straight text detection results

EAST, DeepReg, and R-YOLO have good performance in detecting straight text, but they cannot detect arbitrary-shape text, which is limited in practical applications, and their detection speed is not faster than our model. The experimental results are shown in Table 2. When trained directly on the IC15 dataset, we found that the F-measure of the model reached 79.5%, 1.3 percentage points higher than that of EAST. It also surpassed some models using external datasets for training, such as SSTD, SegLink, and WordSup, and achieved competitive results. After SynthText pre-training, we found that the F-measure of the model increased to 81.5%, surpassing most contrasting models. The inference speed of the model was three times higher than that of R-YOLO (the fastest among all comparison models).

**Table 2:** Text detection results on IC15 and MSRA-TD500, FPS is tested on RTX3090.

Method	External Dataset	IC15				MSRA-TD500			
		Precision	Recall	F-measure	FPS	Precision	Recall	F-measure	FPS
CTPN [7]	×	74.2	51.6	60.9	7.1	-	-	-	-
RRPN [54]	×	82	73	77	-	82	68	74	-
DMPNet [21]	×	73.2	68.2	70.6	-	-	-	-	-
EAST [22]	×	83.6	73.5	78.2	13.2	87.3	67.4	76.1	-
DeepReg [60]	×	82	80	81	-	77	70	74	1.1
PixelLink [30]	×	82.9	81.7	82.3	7.3	81.1	73	76.8	3
**Ours**	×	**88.1**	72.5	79.5	**199.6**	81.9	64.7	72.3	**190.3**
SSTD [61]	PrivateData	80.2	73.9	76.9	7.7	-	-	-	-
SegLink [53]	SynthText	73.1	76.8	75	-	86	70	77	8.9
WordSup [62]	SynthText	79.3	77	78.2	-	-	-	-	-
MCN [63]	SynthText	72	80	76	-	88	79	83	-
R-YOLO [8]	IC17-MLT	87	78.2	82.3	62.5	88.3	76.5	82	40
TextSnake [31]	SynthText	84.9	80.4	82.6	1.1	83.2	73.9	78.3	1.1
**Ours**	SynthText	**84.9**	78.4	81.5	**199.6**	84.2	72.2	77.8	**190.3**

When the model was directly trained on MSRA-TD500, the F-measure was 72.3%. After training with SynthText, the F-measure of the model was improved to 77.8%, 0.8 percentage points higher than that of SegLink trained with external data sets. The interference speed of the model was four times that of R-YOLO (the fastest of all comparison models).

**Fig 6:**
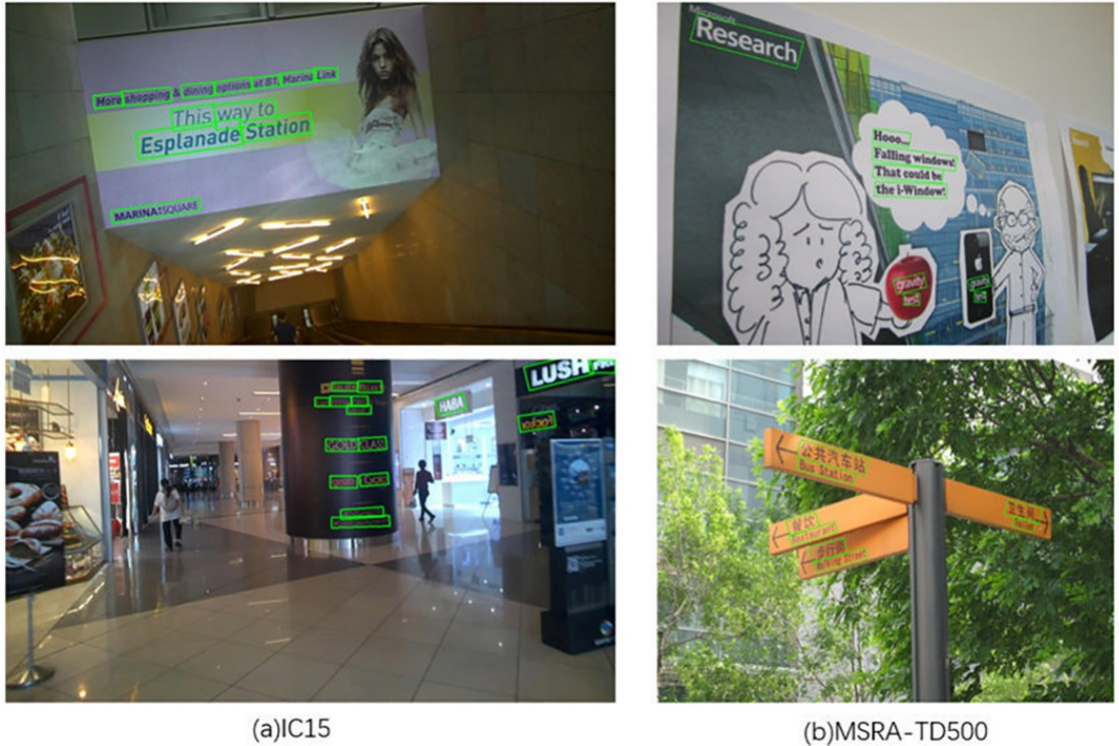
Detection results on IC15 and MSRA-TD500.

In conclusion, our model can also detect Straight Text well. And the detection speed is outstanding. We show some detection results in Fig 6, and it can be seen that our model achieved promising detection results for Straight Text.

### Ablation study

#### Effectiveness of adding branches in the neck

We conducted experiments on the Total-Text dataset and tested the results with three, four, and five branches of the network’s neck with the decoupling output part, as shown in Table 3. The results show that adding branches significantly improved the recall and F-measure of the model. Compared with the three-branch model, the recall of the five-branch model increased by 22.8% and the F-measure increased by 16.1%. Thus, our improvement makes the network more suitable for text detection, which proves the effectiveness of adding branches.

**Table 3:** Experimental results of network neck with a different number of branches on Total-Text.

The number of branches	Total-Text		
	Precision	Recall	F-measure
3	93.2	52.3	67
4	93.4	56.1	70.1
5	93	75.1	83.1

#### Effectiveness of decoupled output

We verified the effectiveness of decoupling output on the IC15 dataset. We conducted experiments on decoupling output and non-decoupling output, under the condition that the neck of the network had five branches. The results are shown in Table 4. The experimental results show that the detection performance of the model was effectively improved after decoupling the output. After decoupling the output, the recall and F-measure of the model increased by 7.3% and 3.7%, respectively, proving that our decoupling operation on the outputs of the network is effective.

**Table 4:** Comparison of results between decoupled and non-decoupled output on IC15, D indicates decoupled outputs and N indicates non-decoupled output.

D/N	IC15		
	Precision	Recall	F-measure
N	90.6	65.2	75.8
D	88.1	72.5	79.5

#### Generalization analysis

To verify the generalization of the model, we conducted cross-dataset experiments, as shown in Table 5, using datasets labeled with the same type as the training and testing sets.

**Table 5:** Cross-datasets experimental results.

Train set	Test set	Precision	Recall	F-measure
SynthText	Total-Text	73.4	41	52.7
SynthText	IC15	68.3	45.7	54.8
Total-Text	IC15	82.1	68.9	74.9
IC15	Total-Text	81.5	60.2	69.3
CTW1500	MSRA-TD500	84.3	66.6	74.4
MSRA-TD500	CTW1500	82.2	67.9	74.3

The experimental results demonstrate that our model performs well on unseen datasets, with an F-measure of over 74% achieved when cross-validated using CTW1500 and MSRA TD500.

#### Loss function analysis

**Fig 7:**
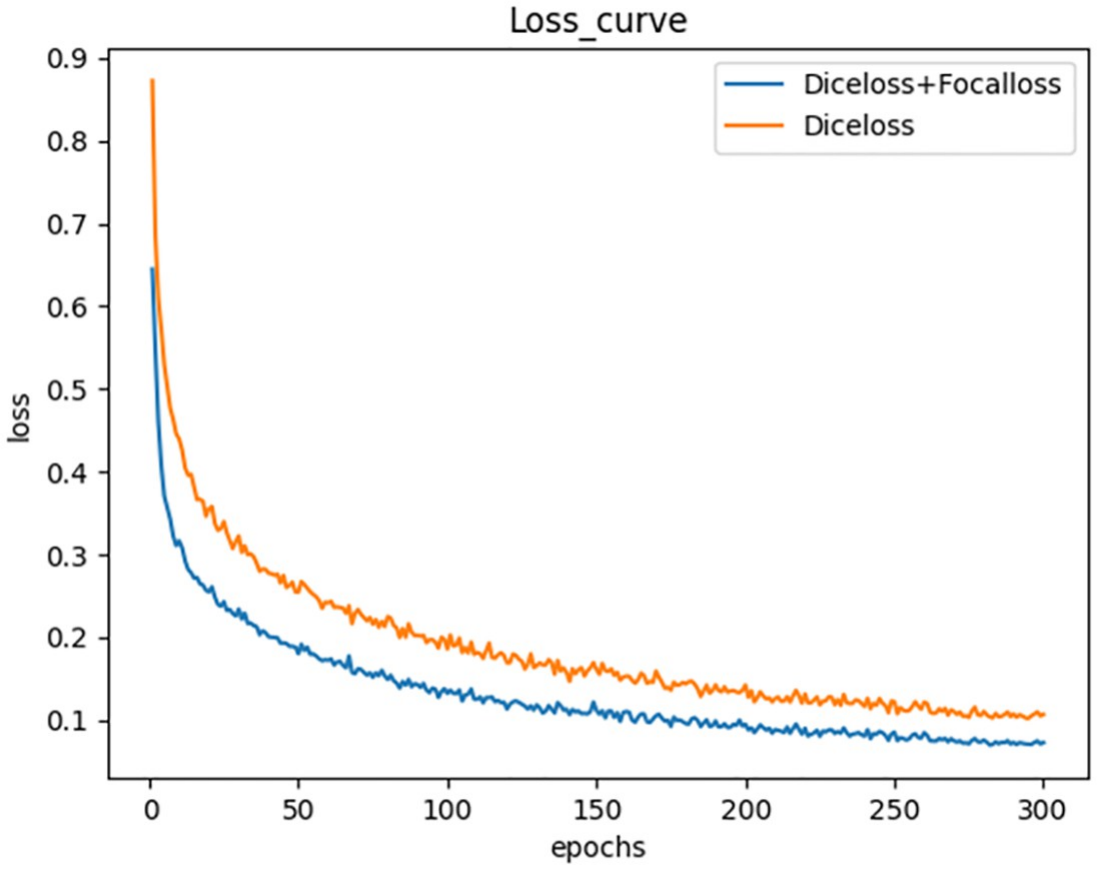
Comparison of loss curves.

We compared the loss curves before and after the change of the loss function on the MSRA-TD500 dataset, as shown in Fig 7. After using the loss function weighted by dice loss and focal loss, the model training is smoother, and the loss decreases faster, which proves that our improvement of the loss function is effective.

**Fig 8:**
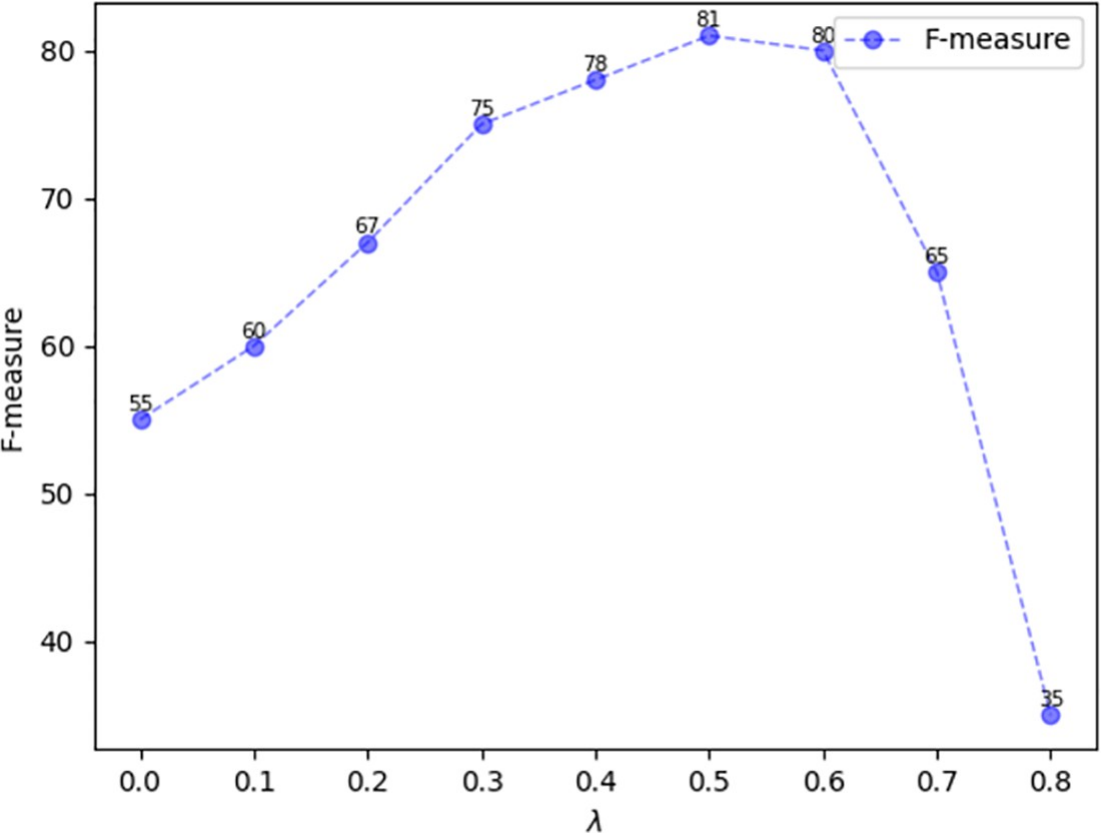
The plot of the variation of λ versus F-measure on IC15.

To verify the effect of parameter *λ* for the model, we took *λ* in the loss function *L*_*tex*_ from 0 to 1 and trained it on the IC15 dataset. The results show that *λ* taken as 0.5 was more favorable for the performance of the model, as shown in Fig 8.

#### Influence of shrinking rate

**Fig 9:**
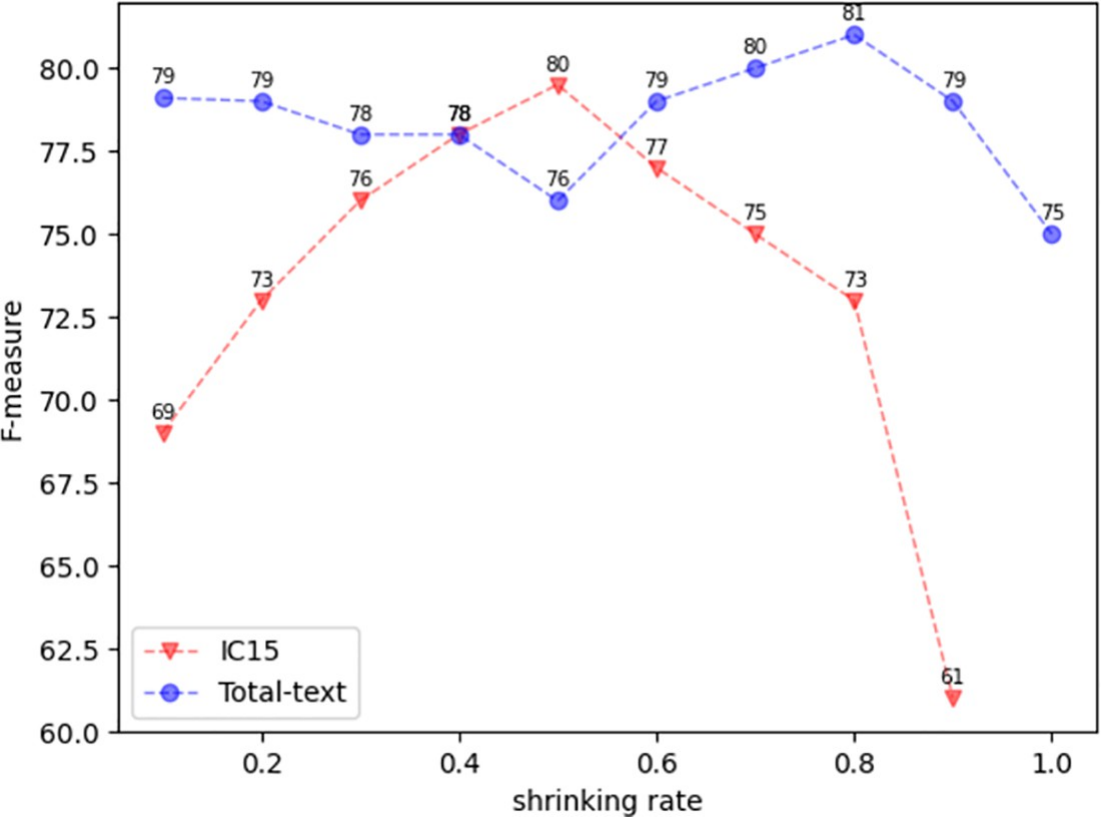
Text detection results under different shrinking rates.

To verify the impact of the shrinking rate on detection results, we conducted experiments using different shrinking rates on the total text and IC15 datasets. As shown in Fig 9, in the total-text dataset, the best detection result is obtained when the shrinking rate is set to 0.8, and the best detection result is obtained when the shrinking rate is set to 0.5 on the ic15 dataset.

## Discussion

Previous experiments have shown that our model can detect arbitrarily shaped text well, with competitive results on different datasets. However, there are some examples of detection failures, such as false detection of text-like areas, mainly due to the lack of relevant training samples. We believe our model can effectively avoid false detection of text-like regions objects when sufficient training samples are available. The detection results are incomplete when there is a large space between text regions. This problem also exists in other good text detection models, which can be solved by including linguistic features.

## Conclusion

In this paper, we proposed an arbitrary-shape text detector that can be trained end-to-end and whose detection speed reaches the real-time level. The contributions of this paper are as follows: 1) We combined the improved YOLOv6 network for text objects with the PA algorithm to realize real-time detection of arbitrary-shape text. 2) We decoupled the output of the PA algorithm in the detection head, which effectively improves the detection performance of the model. 3) We use a loss function weighted by dice loss and focal loss to improve the training performance of the model.

We carried out experiments on Total-Text, CTW1500, ICDAR2015, and MSRA-TD500. The experiments show that our model could effectively detect arbitrary-shape text, including horizontal, directional, and irregular text. Our method achieved an 84.1% F-measure on Total-text and an 81.1% F-measure on CTW1500, surpassing the results of some previous state-of-the-art methods. In particular, the reference speed of our model reached 291.8 FPS and 193.4 FPS on Total-text and CTW1500, respectively, surpassing the results of the previous model. Similarly, the experimental results of the model on IC15 and MSRA-TD500 were also competitive. Future research may add a text recognition end to the model to form an end-to-end text recognition model that can simultaneously achieve real-time detection and recognition of arbitrary-shape texts.
